# Piceatannol as a potential antiviral agent against vaccinia virus at multiple stages

**DOI:** 10.3389/fmicb.2026.1735694

**Published:** 2026-02-05

**Authors:** Liyuan Zhang, Yuwen Liu, Daoqun Li, Junwen Luan, Leiliang Zhang

**Affiliations:** 1 Department of Clinical Laboratory Medicine, The First Affiliated Hospital of Shandong First Medical University and Shandong Provincial Qianfoshan Hospital, Jinan, Shandong, China; 2 Department of Pathogen Biology, School of Clinical and Basic Medical Sciences, Shandong First Medical University and Shandong Academy of Medical Sciences, Jinan, Shandong, China

**Keywords:** extracellular enveloped virus (EEV), mpox virus (MPXV), piceatannol, vaccinia virus (VACV), virucidal

## Abstract

The resurgence of mpox, caused by the mpox virus (MPXV), has intensified the demand for effective antiviral agents. This study evaluates the antiviral activity of piceatannol, a natural polyphenol, utilizing vaccinia virus (VACV) as a model due to the genetic conservation between the two viruses. *In vitro* experiments demonstrated that piceatannol significantly inhibited VACV replication through both extracellular enveloped virus (EEV), and intracellular mature virus (IMV), with median effective concentrations (EC_50_) of 76.27 μM, and 63.93 μM, respectively. Further analysis revealed that piceatannol also reduced VACV entry and replication in HeLa cells. Molecular docking studies revealed a stable interaction between piceatannol and the palmitoylated F13 protein of VACV, with a calculated binding energy of−19.06 kcal/mol. This interaction suggests a mechanism by which piceatannol inhibits the generation of EEV. Additionally, piceatannol effectively limited the spread of VACV in cell cultures and exhibited significant virucidal effects on both the IMV and EEV forms. Collectively, our findings suggest that piceatannol holds promise as a therapeutic agent against orthopoxviruses infections by modulating key viral processes, warranting further exploration in clinical settings.

## 1 Introduction

The global outbreak of mpox has drawn significant attention in recent years. Mpox, a zoonotic infectious disease caused by the mpox virus (MPXV), was primarily prevalent in Central and West Africa throughout the 1970's, with the first outbreak in the United States occurring in 2003 (Reed et al., 2004). However, it was largely overlooked at that time. It was not until 2022 that multiple countries began reporting confirmed cases of MPXV, raising global concerns as the outbreak rapidly expanded (Nuzzo et al., 2022). Vaccinia virus (VACV), the prototypical poxvirus used for smallpox eradication, belongs to the same family as the MPXV and is genetically highly conserved. Members of the *Orthopoxvirus* genus produce two distinct infectious particles: intracellular mature virus (IMV) and extracellular enveloped virus (EEV) (Locker et al., 2000). F13 is the most abundant component of the EEV membrane and plays a pivotal role in the life cycle of orthopoxviruses, particularly in the encapsulation of IMV into EEV. F13 has been identified as a target for the antiviral drug tecovirimat (also known as ST-246) (Li et al., 2025). This drug alters F3's localization, affecting its structure and function, and effectively inhibits the production and release of enveloped virions in infected cells (Merchlinsky et al., 2019). Recent studies indicate that tecovirimat functions as molecular glue, promoting F13 oligomerization and thereby inhibiting poxvirus (Vernuccio et al., 2025).

Previously, researchers utilized molecular dynamics simulations to investigate the interactions between the MPXV F13 protein and natural polyphenols (Rout et al., 2023). By calculating binding free energies, they identified that demethoxycurcumin, ellagic acid, piceatannol, and myricetin could associate MPXV F13 protein (Rout et al., 2023). Natural polyphenols are notable for their clinical usability, low production costs, ease of preparation, and minimal side effects, showing strong therapeutic potential, and antiviral activity, making them a focus of research. Given that VACV and MPXV both belong to the *Orthopoxvirus* genus, and are genetically highly conserved, and considering that VACV was previously used as a smallpox vaccine with high safety (Jacobs et al., 2009), this study investigates the anti-MPXV activity of natural polyphenols using VACV.

## 2 Materials and methods

### 2.1 Cells, viruses, and reagents

HeLa, HEK-293 and BSC-1 cell lines are bought from Cobioer Biosciences Co., LTD. Huh7.5.1 cell line is a gift from Dr. Francis Chisari in the Scripps Research Institute. HeLa, HEK-293, Huh7.5.1, and BSC-1 cell lines were maintained in Dulbecco's modified Eagle's media (DMEM) supplemented with 10% fetal bovine serum (FBS), 1x L-Glutamine, and Penicillin/Streptomycin at 37 °C in a 5% CO_2_ atmosphere. The WR strain of (VACV) and the recombinant VACV A4-YFP, an engineered variant containing an YFP cassette, were kept in our lab and propagated in HeLa cells. Piceatannol (HY-13518), demethoxycurcumin (HY-N0006), ellagic acid (HY-B0183), and myricetin (HY-15097) were purchased from MedChemExpress and dissolved in dimethyl sulfoxide (DMSO) to a stock concentration of 400 mM.

### 2.2 Cell viability

Cell viability was assessed using a Cell Counting Kit-8 (CCK-8) (PF00004, Proteintech, USA). Approximately 3,000 cells were seeded per well in 96-well plates containing DMEM with 10% FBS and incubated for 24 h. The cells were then treated with piceatannol at concentrations of 10 μM, 20 μM, 40 μM, 80 μM, 160 μM, 320 μM, and 400 μM for an additional 24 h. After incubation, 10 μL of CCK-8 reagent was added to each well, and the cells were incubated for another hour. The OD was measured at 450 nm using a TECAN SPARK instrument. The cell viability curve was presented as a percentage of the OD value of treated samples relative to that of untreated controls.

### 2.3 Plaque assays

Confluent BSC-1 monolayers were inoculated with serial dilutions of collected supernatants and cells, performed in duplicate wells. Following a 1.5-h incubation with the viruses, the inoculum was removed, and the cells were washed twice with PBS. Subsequently, 10% FBS DMEM was added. After 60 h, cells were stained with 0.1% crystal violet for 10 to 20 min. The crystal violet was then discarded, and wells were washed twice with PBS, allowing them to dry. Viral titers were determined by counting plaques, requiring 20 to 100 visible plaques for accurate measurements.

### 2.4 Prediction of the interaction between the MPXV F13 protein with piceatannol

First, we utilized the online tools CSS-Palm (http://csspalm.biocuckoo.org/) and GPS-Palm (http://gpspalm.biocuckoo.cn/) to predict palmitoylation sites on the MPXV F13 protein. Based on reported structural predictions, we performed homology modeling using the AlphaFold3 web server to obtain the three-dimensional structure of the palmitoylated MPXV F13 protein. This model was then superimposed onto the predicted structure of the MPXV F13 protein, and model quality was assessed using root-mean-square deviation (RMSD) by Discovery Studio 2016 or Ramachandran plots by SAVES v6.0. Finally, we employed Discovery Studio 2016 to conduct blind docking simulations between piceatannol, and the palmitoylated MPXV F13 protein.

### 2.5 Acyl-biotin exchange (ABE) assay

To assess the palmitoylation level of MXPV F13-HA, this study utilized the ABE assay. The cell lysis buffer was formulated with 50 mM Tris-HCl (pH 7.5) (T1140, Solarbio, Beijing, China), 150 mM NaCl, 10% glycerol, 1% IGEPAL CA-630 (I3021, Sigma-Aldrich, St. Louis, MO, USA), and supplemented with a protease inhibitor mixture (100 × , Beyotime, P1010), 0.1 mM PMSF (ST506, Beyotime, Beijing, China), and 50 mM N-ethylmaleimide (NEM) (E100553, Aladdin, Shanghai, China). Following lysis of cells transfected with a plasmid encoding MXPV F13-HA, NEM was employed to pre-block free thiol groups in the proteins. Immunoprecipitation was then performed using a mouse HA antibody (Beijing Xuheyuan Biotech Co., Ltd., XHY168L). The resulting precipitated complexes were treated with hydroxylamine (HAM) (H828371, MACKLIN, Shanghai, China) to liberate palmitoylated cysteine residues, which were subsequently labeled with biotin-BMCC (B2112, ProteoChem, Shanghai, China). Finally, the biotin labeling signals were detected via Western blotting using streptavidin-HRP (M00091, GenScript, Nanjing, Jiangsu, China), indicating the palmitoylation level of MXPV F13-HA. Detection of F13-HA was carried out using rabbit anti-HA (Beijing Xuheyuan Biotech Co., Ltd., XHY022L).

### 2.6 Immunofluorescence assay

BSC-1 cells were infected with A4-YFP virus for 1h and then were treated with piceatannol at concentrations of 20 μM, 40 μM, 80 μM, and 160 μM, or 0.04% DMSO for 24 h. Cells in six-well plates were fixed with methanol at −20 °C for 10 min before imaging by Olympus IX73P2F microscope (Japan).

### 2.7 Viral RNA extraction and quantitative real-time RT-PCR

Total RNA was extracted using the FastPure Cell/Tissue Total RNA Isolation Kit V2 (Vazyme, Nanjing, China). Reverse transcription was performed with the PrimeScript™ II 1st Strand cDNA Synthesis Kit (Takara Bio). The synthesized cDNA was analyzed via quantitative real-time PCR using TB Green Premix Ex Taq™ II (Tli RNaseH Plus) (Takara Bio) in a LightCycler 480 System (Roche). Relative mRNA values were calculated using the 2^−Δ*ΔCT*^ method, with β-actin serving as an internal control in each sample. The primers used were as follows: E9L forward 5′-CGGCTAAGAGTTGCACATCCA-3′, and reverse 5′-CTCTGCTCCATTTAGTACCGATTCT-3′, while β-actin primers were forward 5′-GATCCACATCTGCTGGAAG-3′, and reverse 5′-CAGCACAATGAAGATCAAGA-3′. Each sample was assayed in triplicate.

### 2.8 Statistical analysis

All results were consistent and reproducible across at least three independent experiments. Data are presented as means ± standard error of the mean (SEM). GraphPad Prism 9 software was utilized for statistical analysis. The following *P*-values were considered indicative of statistical significance: ^*^P < 0.05; ^**^P < 0.01; ^***^P < 0.001; and ^****^P < 0.0001.

## 3 Results

### 3.1 Piceatannol inhibits VACV

**Figure 1:**
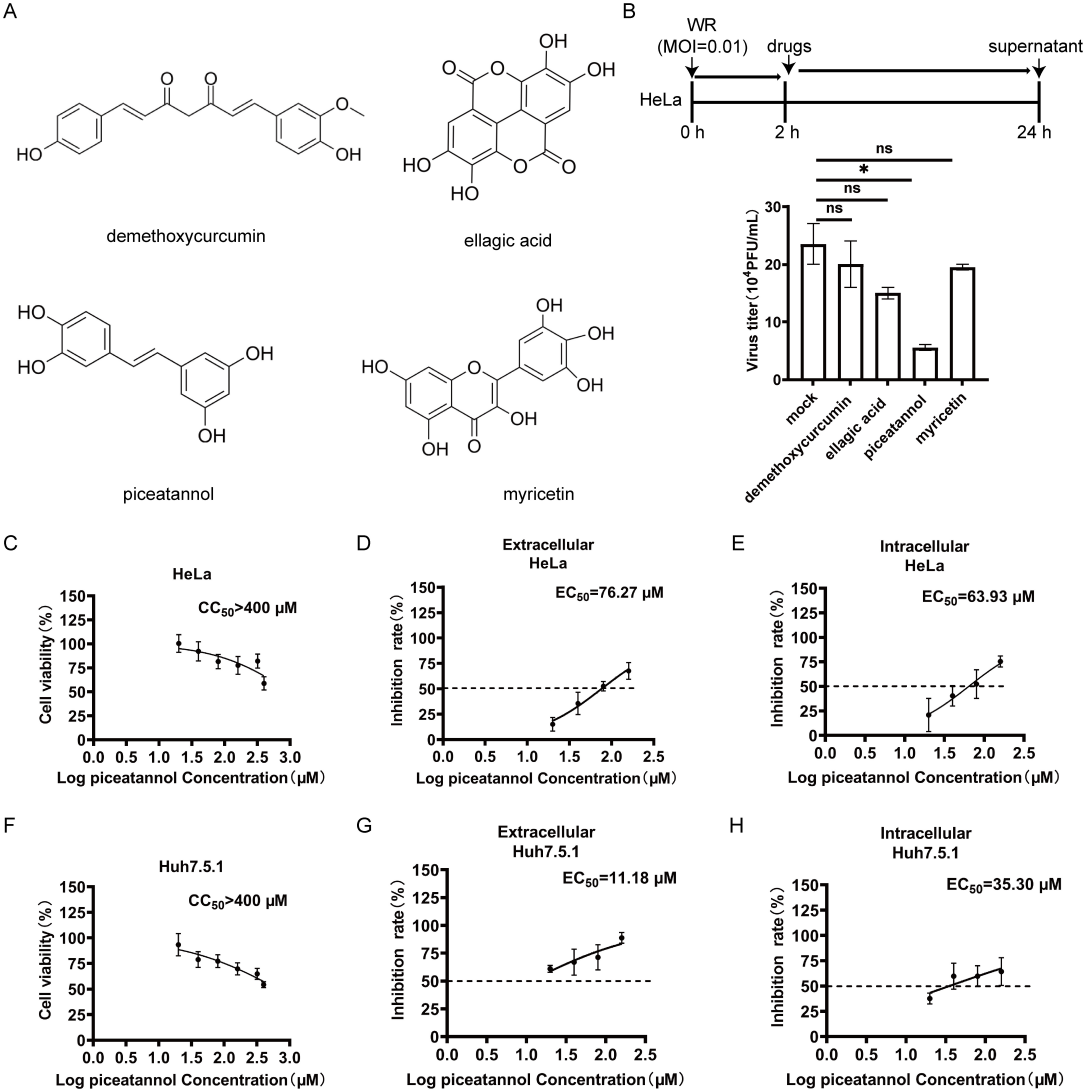
Inhibition of VACV by piceatannol. **(A)** The chemical structures of the four compounds. **(B)** The inhibitory effects of the four compounds on VACV. HeLa cells were first infected with VACV WR at an MOI of approximately 0.01 for 2 h. Subsequently, the cells were treated for 22 h with demethoxycurcumin, ellagic acid, piceatannol, and myricetin at a concentration of 20 μM, or mock treatment (medium only). The supernatants were collected for titer. **(C)** HeLa cells were treated with piceatannol at concentrations of 10 μM, 20 μM, 40 μM, 80 μM, 160 μM, 320 μM, and 400 μM, or with 0.04% DMSO, for a duration of 24 h, followed by the addition of CCK-8 reagent. **(D and E)** The inhibitory effect of piceatannol on VACV in HeLa cells. HeLa cells were infected with VACV WR at an MOI of approximately 3 for 2 h. Afterward, the cells were treated with piceatannol at concentrations of 20 μM, 40 μM, 80 μM, and 160 μM, or with 0.04% DMSO, for a duration of 18 h, and then supernatants **(D)** and cells **(E)** were collected and titrated using BSC-1 cells. **(F)** Huh7.5.1 cells were treated with piceatannol at concentrations of 10 μM, 20 μM, 40 μM, 80 μM, 160 μM, 320 μM, and 400 μM, or with 0.04% DMSO, for a duration of 24 h, followed by the addition of CCK-8 reagent. **(G and H)** The inhibitory effect of piceatannol on VACV in Huh7.5.1 cells. Huh7.5.1 cells were infected with VACV WR at an MOI of approximately 3 for 2 h. Then, the cells were treated with piceatannol at concentrations of 20 μM, 40 μM, 80 μM, and 160 μM, or with 0.04% DMSO, for a duration of 18 h, and supernatants **(G)** and cells **(H)** were collected and titrated using BSC-1 cells. *P < 0.05. “ns” indicates no statistical significance.

To investigate the antiviral effects of demethoxycurcumin, ellagic acid, piceatannol, and myricetin (Figure 1A). HeLa cells were infected with VACV WR at a MOI of approximately 0.01. The results showed that 20 μM of piceatannol significantly reduced the viral titer in the supernatant compared to the other three compounds (Figure 1B). This finding confirms that piceatannol has stronger inhibitory effects on VACV, establishing it as the focus of our experiments. To evaluate the influence of piceatannol on cell viability, HeLa cells were treated with various concentrations of piceatannol, and cell viability was assessed using CCK-8 assays. The results showed that the half cytotoxic concentration (CC_50_) of piceatannol in HeLa cells was above 400 μM (Figure 1C). To further investigate the inhibitory effects of piceatannol on VACV, HeLa cells were infected with VACV WR at an MOI of approximately 3 for 2 h, followed by treatment with different concentrations of piceatannol for 18 h. Supernatants and cells were then collected, and viral titers were measured using BSC-1 cells. The median effective concentration (EC_50_) of piceatannol against extracellular VACV (mainly EEV) in HeLa cells was found to be 76.27 μM, while the EC_50_ against intracellular VACV (mainly IMV) was 63.93 μM (Figures1D, 1E). Similarly, Huh7.5.1 cells were treated with varying concentrations of piceatannol to assess cell viability, resulting in a CC_50_ above 400 μM (Figure 1F). Following infection with VACV WR at an MOI of approximately 3 for 2 h, Huh7.5.1 cells were treated with different concentrations of piceatannol for 18 h. The results showed that the EC_50_ of piceatannol against extracellular VACV (mainly EEV) in Huh7.5.1 cells was 11.18 μM, and the EC_50_ against intracellular VACV (mainly IMV) was 35.30 μM (Figures1G, 1H). Table 1 summarized the inhibition of VACV by piceatannol in HeLa and Huh7.5.1 cells. Collectively, these results indicate that piceatannol's inhibitory effect on VACV is dose-dependent and affects both EEV and IMV.

**Table 1:** Summary of inhibition of vaccinia virus by piceatannol in HeLa and Huh7.5.1 cells.

**Cell line**	**EC_50_**	**CC_50_**	**SI (CC_50_/EC_50_)**
HeLa	76.27 μM for extracellular virus	>400 μM	>5.24
	63.93 μM for intracellular virus		>6.26
Huh7.5.1	11.18 μM for extracellular virus		>35.78
	35.30 μM for intracellular virus		>11.33

### 3.2 Piceatannol inhibits the VACV entry and replication

**Figure 2:**
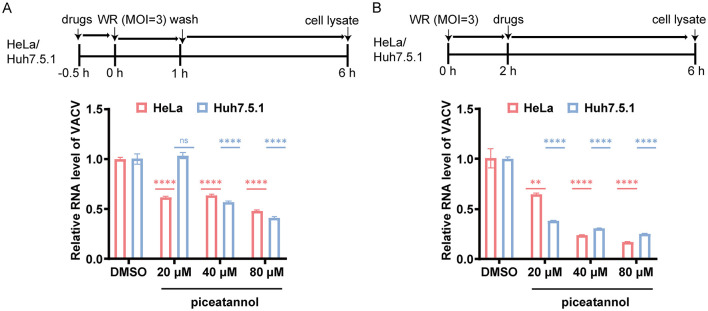
Piceatannol inhibits the VACV entry and replication. **(A)** The inhibitory effect of piceatannol on VACV invasion. HeLa and Huh7.5.1 cells were treated with piceatannol at concentrations of 20 μM, 40 μM, and 80 μM, or with 0.02% DMSO, for a duration of 0.5 h. Subsequently, the cells were infected with VACV WR at an MOI of approximately 3 for 1 h, followed by treatment with fresh culture medium for 5 h before extracting total cellular RNA. **(B)** The inhibitory effect of piceatannol on VACV replication. HeLa and Huh7.5.1 cells were infected with VACV WR at an MOI of approximately 3 for 2 h. Afterward, the cells were treated with piceatannol at concentrations of 20 μM, 40 μM, and 80 μM, or with 0.02% DMSO, for a duration of 4 h before extracting total cellular RNA. In all experiments, the relative expression levels of E9L were measured using quantitative real-time PCR. **P < 0.01; ****P < 0.0001. “ns” indicates no statistical significance.

To explore piceatannol's early effects on VACV entry, we treated HeLa cells with varying concentrations of piceatannol for 0.5 h, followed by infection with VACV WR at an MOI of about 3 for 1 h. The drug and viral mixture was discarded, and fresh medium was added for an additional 5 h before measuring the RNA levels of intracellular virus. The results showed that, compared to the DMSO group, the levels of viral RNA were reduced after treatment with piceatannol at concentrations of 20 μM, 40 μM, and 80 μM, indicating that piceatannol inhibits VACV entry (Figure 2A). Similar experiments in Huh7.5.1 cells yielded comparable results. Viral RNA levels remained unchanged at 20 μM but decreased at 40 μM and 80 μM (Figure 2A). These findings suggest that piceatannol could inhibit VACV entry.

To further verify piceatannol's effect on VACV replication, HeLa cells were infected with VACV WR at an MOI of 3 for 2 h. Subsequently, cells were treated with varying concentrations of piceatannol for 4 h before assessing intracellular viral RNA levels. The results indicated that, compared to the DMSO group, viral RNA levels decreased in a dose-dependent manner after piceatannol treatment, demonstrating that piceatannol can inhibit VACV replication (Figure 2B). Similarly, in Huh7.5.1 cells, a corresponding decline in viral RNA levels was observed (Figure 2B). Collectively, these results provide evidences that piceatannol exerts inhibitory effects during the early stages of the VACV life cycle.

### 3.3 Piceatannol inhibits the generation of VACV EEV by regulating F13

**Figure 3:**
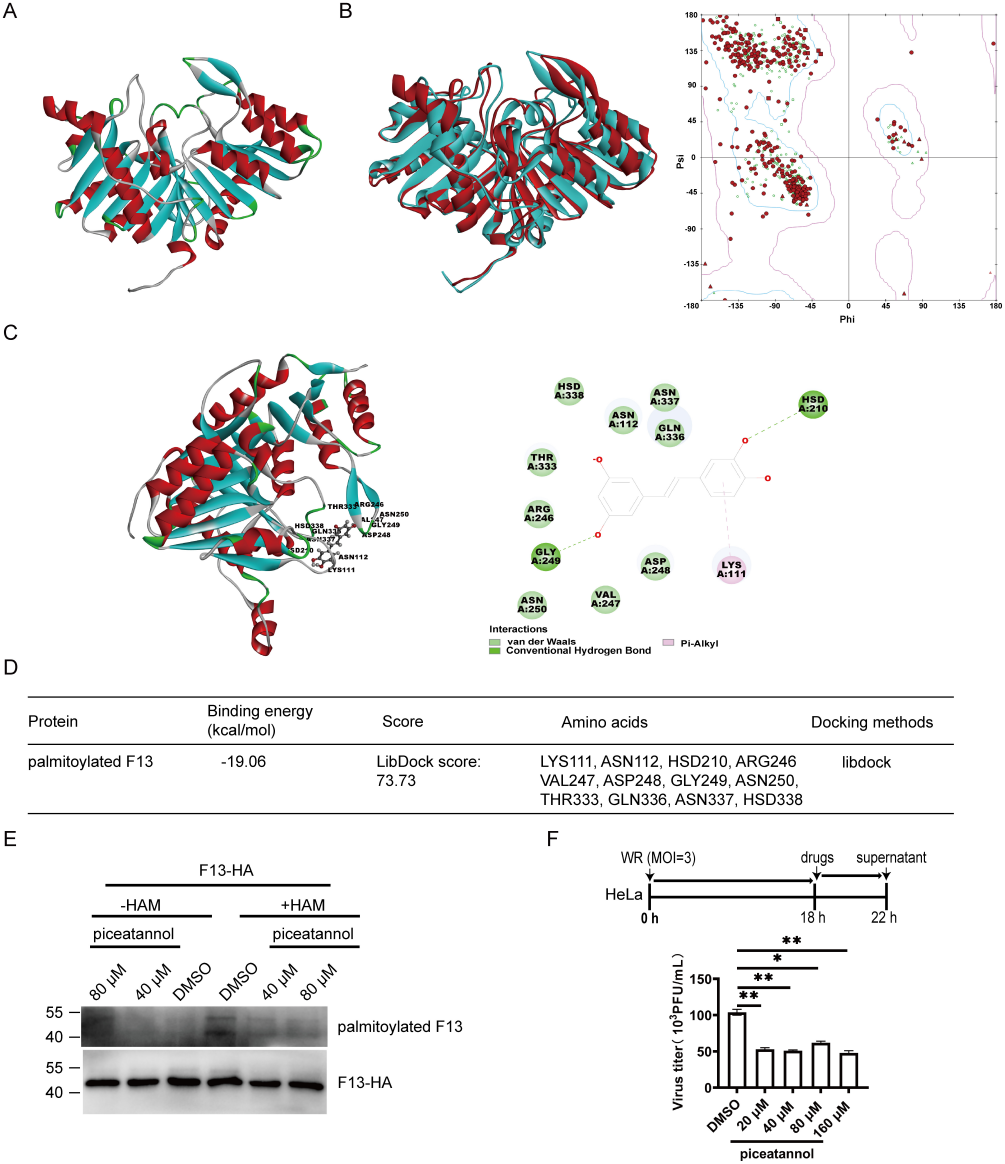
Piceatannol inhibits the generation of VACV EEV by regulating F13. **(A)** Prediction of the 3D structure of palmitoylated F13 protein using the AlphaFold3 web server. Red indicates α-helices, blue indicates β-sheets, and green indicates β-turns. **(B)** Left panel: Superposition of MPXV F13 protein and palmitoylated F13 protein; Right panel: Ramachandran plot of palmitoylated F13 protein (red points). **(C)** Docking results of palmitoylated F13 with piceatannol, highlighting interacting amino acids and the types of interactions. **(D)** Docking results of palmitoylated F13 from this study. **(E)** Piceatannol reduces the palmitoylation of MPXV F13. In HEK-293T cells, MPXV F13-HA was transfected, and the medium was changed after 6 h. The cells were then treated with piceatannol at concentrations of 40 μM and 80 μM, or with 0.02% DMSO, for a duration of 12 h. Subsequently, the ABE experiment was performed, and detection was carried out using western blot. **(F)** The effect of piceatannol on VACV EEV generation. HeLa cells were infected with VACV WR at an MOI of approximately 3 for 18 h. Subsequently, the cells were treated with piceatannol at concentrations of 20 μM, 40 μM, 80 μM, and 160 μM, or with 0.04% DMSO, for a duration of 4 h, after which the supernatant was collected. Finally, viral titers were measured using BSC-1 cells. *P < 0.05; **P < 0.01. “ns” indicates no statistical significance.

Since the F13 protein of VACV undergoes palmitoylation, we investigated whether F13 associates with palmitoylated F13. We predicted the 3D structure of the palmitoylated F13 protein using the amino acid sequence of the F13 protein from MPXV through Discovery Studio and the AlphaFold3 web server (Figure 3A). To validate the accuracy of the predicted structure, we superimposed it with the known structure of the MPXV F13 protein. The comparison revealed a high degree of similarity between the predicted and known structures, confirming the reliability of our predictions. Additionally, analysis via the Ramachandran plot indicated that most amino acid residues in the predicted structure fall within acceptable regions, further validating the prediction's accuracy (Figure 3B). Next, we explored the interactions between palmitoylated F13 and piceatannol through molecular docking studies. The docking results indicated that piceatannol binds stably to a specific region of the palmitoylated F13 protein, forming a stable complex (Figure 3C). An analysis of the interacting amino acids and forces revealed that piceatannol primarily interacts through hydrogen bonds and hydrophobic interactions with several residues of palmitoylated F13, including LYS111, ASN112, HSD210, and ARG246, among others (Figure 3C). The docking results further indicated a binding energy of −19.06 kcal/mol between piceatannol and palmitoylated F13 (Figure 3D). This negative value suggests a significant binding affinity, indicating that piceatannol may inhibit orthopoxviruses, including MPXV.

To investigate whether piceatannol affects the palmitoylation of the MPXV F13 protein, HEK-293T cells were transfected with the plasmid MPXV F13-HA. After 6 h, the medium was changed, and the cells were treated with piceatannol at concentrations of 40 μM, 80 μM, or 0.02% DMSO for 12 h. The palmitoylation levels were then assessed using the ABE assay. The results indicated that treatment with piceatannol at both 40 μM and 80 μM reduced the palmitoylation levels of the MPXV F13 protein (Figure 3E). This finding suggests that piceatannol may inhibit the poxvirus by inhibiting the palmitoylation of the F13 protein.

To further validate the effect of piceatannol on VACV EEV production, HeLa cells were infected with VACV WR at an MOI of approximately 3 for 18 h. The cells were then treated with different concentrations of piceatannol for 4 h, followed by measurement of viral titers in the supernatant. The results demonstrated that the viral titers in the supernatants of piceatannol-treated cells decreased by 50% compared to the DMSO control group, indicating that piceatannol could effectively inhibit VACV EEV generation (Figure 3F). Based on both our predictive and experimental results, we speculated that piceatannol may inhibit VACV EEV generation by regulating the F13 protein.

### 3.4 Piceatannol inhibits the spread of VACV

**Figure 4:**
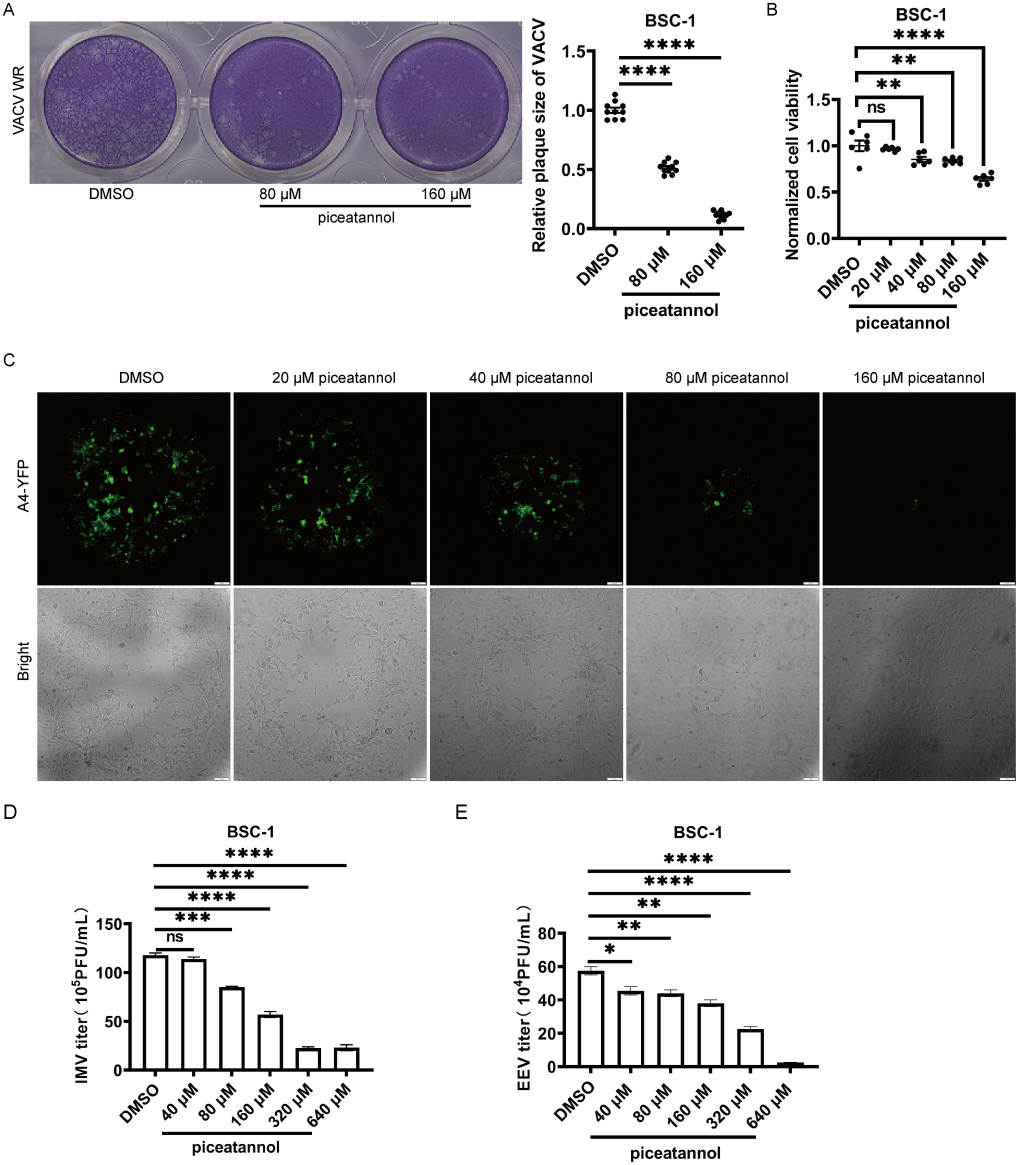
Piceatannol inhibits the spread of VACV and exhibits virucidal effects against VACV. **(A)** The inhibitory effect of piceatannol on VACV plaque size. BSC-1 cells were infected with VACV WR for 2 h and then treated with piceatannol at concentrations of 80 μM and 160 μM, or with 0.04% DMSO, for a duration of 46 h. **(B)** The effect of piceatannol on cell viability at 48 h. BSC-1 cells were treated with piceatannol at concentrations of 20 μM, 40 μM, 80 μM, and 160 μM, or with 0.04% DMSO, for a duration of 48 h. **(C)** The inhibitory effect of piceatannol on VACV fluorescence area. BSC-1 cells were infected with A4-YFP at a PFU of approximately 200 for 1 h. The cells were then treated with piceatannol at concentrations of 20 μM, 40 μM, 80 μM, and 160 μM, or with 0.04% DMSO, for a duration of 24 h. Observations were made using Olympus IX73P2F microscope. Scale bar: 100 μm. **(D)** The virucidal effect of piceatannol on VACV IMV. Piceatannol at concentrations of 40 μM, 80 μM, 160 μM, and 320 μM, or 0.08% DMSO, were incubated with IMV of VACV WR in a 37 °C incubator for 0.5 h. The mixture was then diluted and added to BSC-1 cells for plaque assay. **(E)** The virucidal effect of piceatannol on VACV EEV. Piceatannol at concentrations of 40 μM, 80 μM, 160 μM, and 320 μM, or 0.08% DMSO, were incubated with EEV of VACV WR in a 37 °C incubator for 0.5 h. The mixture was then diluted and added to BSC-1 cells for plaque assay. *P < 0.05; **P < 0.01; ***P < 0.001; ****P < 0.0001. “ns” indicates no statistical significance.

Next, to explore the effects of piceatannol on the later stages of the VACV life cycle, BSC-1 cells were infected with VACV WR (PFU approximately 20–200) for 2 h. Subsequently, the cells were treated with piceatannol at concentrations of 80 μM and 160 μM, or with 0.04% DMSO, for a duration of 46 h. The results demonstrated that the area of viral plaques formed was significantly reduced in the piceatannol-treated groups compared to the DMSO group (Figure 4A). Examination of the effects of piceatannol on BSC-1 cell viability revealed that at 160 μM, cell viability did not fall below 50% (Figure 4B). This indicates that the inhibition of plaque size observed is solely attributable to piceatannol itself. Additionally, since A4-YFP (a VACV strain tagged with YFP) possesses intrinsic YFP fluorescence, it allows for a visual observation of viral changes within cells. BSC-1 cells were infected with A4-YFP (PFU approximately 20–200) for 1 h, followed by treatment with varying concentrations of piceatannol for 24 h, and then observed under an inverted fluorescence microscope. The results showed that, compared to the DMSO group, the fluorescent area decreased with increasing piceatannol concentrations of 40 μM, 80 μM, and 160 μM (Figure 4C). These results collectively indicate that piceatannol effectively inhibits the release of VACV and its intercellular spread.

### 3.5 Piceatannol exhibits virucidal effects against VACV

To investigate the virucidal effects of piceatannol on VACV, VACV WR was co-incubated with varying concentrations of piceatannol at approximately MOI of 0.01 at 37 °C for 0.5 h. Afterward, the mixture was diluted and added to BSC-1 cells, where it was treated for 1.5 h before discarding the virus and drug mixture and adding fresh complete medium for another 60 h. The results revealed that at concentrations of 80 μM and higher, the number of plaques significantly decreased, indicating that piceatannol has virucidal activity against VACV, predominantly in IMV form (Figure 4D). Similarly, using the same method with VACV primarily in EEV form, it was found that treatment with piceatannol at concentrations of 40 μM and above also reduced the number of plaques, further confirming the virucidal effects of piceatannol on VACV in EEV form (Figure 4E). These results demonstrate that piceatannol exhibits significant virucidal activity against VACV.

## 4 Discussion

Piceatannol, a natural polyphenol found abundantly in edible fruits and vegetables such as grapes, mushrooms, blueberries, and passion fruit (Kim et al., 2008), exhibits a wide range of biological activities, including anti-inflammatory, antioxidant, anti-apoptotic, anti-proliferative, and anti-cancer properties (Zhu et al., 2020). Notably, piceatannol has demonstrated antiviral effects by reducing viral gene transcription levels, alleviating apoptosis induced by porcine reproductive, and respiratory syndrome virus (PRV), and increasing serum levels of IL-4, TNF-α, and IFN-γ, thereby providing protective effects against PRV (Wang et al., 2023). Its antiviral mechanism against influenza a virus (IAV), both *in vitro*, and *in vivo*, primarily involves blocking membrane fusion through interaction with hemagglutinin (Huang et al., 2023). Furthermore, piceatannol's inhibition of human cytomegalovirus (hCMV) infection is linked to the suppression of hCMV-induced senescence and the activation of molecular pathways contributing to reactive oxygen species (ROS) production (Wang et al., 2020). Piceatannol is also recognized as an inhibitor of Syk kinase, a non-receptor tyrosine kinase that regulates various cellular processes, including innate immune signaling (Oliver et al., 1994; Pfeiffer and Oliver, 1994). Research indicates that Syk suppresses innate immunity during the later stages of IAV infection, facilitating viral replication; conversely, Syk deficiency enhances the expression of type I and III interferons and inhibits IAV replication, thus rendering mice more resistant to IAV infection (Li et al., 2022). Given the compelling evidence of piceatannol's antiviral mechanisms against various viruses, future studies will aim to elucidate the detailed pathways through which piceatannol inhibits poxviruses. By analyzing these mechanisms, we hope to better understand how piceatannol can be leveraged to combat poxvirus infections and potentially inform the development of novel therapeutic strategies.

In summary, piceatannol exhibits a range of inhibitory effects on VACV, demonstrating its capacity to not only directly kill the virus but also to impede viral entry, replication, EEV production, and VACV dissemination. While tecovirimat is known to inhibit the generation of EEV, piceatannol shows inhibitory effects across multiple stages of the VACV life cycle. As a result, piceatannol appears to be a more promising therapeutic option for orthopoxviruses infections. Our findings highlight the potential of piceatannol as a therapeutic agent against Orthopoxvirus infections.

## Data Availability

The original contributions presented in the study are included in the article/supplementary material further inquiries can be directed to the corresponding author.
